# Operationalizing Multisectoral Nutrition Governance: Examining Commitment, Capacity, and Mechanisms for Action in Scaling Up Nutrition Actions in Kenya

**DOI:** 10.1016/j.cdnut.2026.109403

**Published:** 2026-06-20

**Authors:** Jacob K Korir, Wilna Oldewage-Theron, Malinda J Colwell, Wanjiku N Gichohi-Wainaina

**Affiliations:** 1 Department of Nutritional Sciences, Department of Human Development and Family Sciences, Texas Tech University, Lubbock, TX, United States; 2 Department of Sustainable Food Systems and Development, The University of the Free State, Park West, Bloemfontein, South Africa; 3 WorldFish, Bayan Lepas, Penang, Malaysia

**Keywords:** Multisectoral Nutrition Governance, multisectoral nutrition approaches, nutrition policy, nutrition programming, nutrition actions, Kenya

## Abstract

**Background:**

Effective Multisectoral Nutrition Governance (MNG) is critical for addressing the complex determinants of malnutrition. However, evidence on how key governance functions influence governance outcomes over time remains limited, particularly in low- and middle-income countries.

**Objectives:**

This study assessed the status, evolution, and drivers of MNG in Kenya from 2012 to 2023. It examined how commitment, capacity, and mechanisms for action explain current MNG status and perceived improvements over time.

**Methods:**

A retrospective cross-sectional study was conducted using a validated MNG assessment tool. Respondents assessed current governance conditions in 2023 and retrospectively for 2012. Quantitative data were collected from 112 respondents across government and nongovernmental sectors. Descriptive statistics were used to assess governance trends. Wilcoxon Signed-Rank Tests and multiple linear regression were used to examine changes in MNG over time and identify factors associated with MNG outcomes. Relative importance analysis quantified the contribution of each governance theme.

**Results:**

A total of 96 out of 112 respondents completed the survey. MNG status improved significantly from 58.4% in 2012 to 71.1% in 2023 (*P* < 0.01). Significant improvements were also observed across MNG themes; commitment (*r*_*b*_ = 0.68), capacity (*r*_*b*_ = 0.61), and mechanisms for action (*r*_*b*_ = 0.67). Relative importance analysis indicated that capacity accounted for the largest share of explained variance (35.5%), followed by mechanisms for action (33.2%) and commitment (31.2%). However, the overlapping confidence intervals indicated that all 3 themes were comparably associated with variations in MNG status and improvement over time.

**Conclusions:**

Kenya has made significant progress in strengthening MNG over the past decade. Although commitment and mechanisms for action remain essential, the capacity theme reflects the operational conditions through which governance improvements are implemented. Continued investments in institutional and operational capacity, including predictable and sustainable financing, are therefore important for sustaining and consolidating governance gains.

## Introduction

Global progress in reducing malnutrition is off track, with about 673 million people experiencing chronic hunger in 2024 and more than 2 billion affected by at least one form of malnutrition, including micronutrient deficiencies, overweight, or obesity [1,2]. These persistent and multidimensional burdens reflect structural drivers such as poverty, unaffordable healthy diets arising from dysfunctional food systems, and weak health systems unable to deliver nutrition services at scale. This underscores that malnutrition is a systemic challenge requiring sustained, coordinated, and multisectoral strategies to effectively address the complex determinants of malnutrition [[3], [4], [5]]. Despite socioeconomic progress, Kenya faces a persistent dual burden of malnutrition, with ongoing child undernutrition and micronutrient deficiencies alongside rapidly rising overweight, obesity, and diet-related noncommunicable diseases [[6], [7], [8]]. Although stunting declined from 35% (2009) to 18% (2022), large rural, socioeconomic, and demographic inequalities remain, and micronutrient deficiencies affect at least half of women and children. These overlapping burdens undermine human capital and are estimated to reduce national GDP by up to 6% annually [[9], [10], [11]].

Multisectoral Nutrition Governance (MNG) is increasingly recognized as a crucial mechanism for scaling effective nutrition actions and achieving sustainable outcomes particularly in low- and middle-income countries [4,12]. MNG refers to the institutional processes through which policies are formulated, implemented, and monitored through coordinated action across sectors and levels of governance. It encompasses six interrelated domains: political commitment and leadership, policy coherence and coordination, transparency and accountability, sustainable financing, implementation capacity, and results monitoring [4,13,14]. Effective MNG ensures that nutrition policies are prioritized, adequately funded, and implemented with strong accountability mechanisms [12,14,15]. In Kenya, the commitment to global goals and nutrition targets, adoption of various policy initiatives, including the Kenya Nutrition Action Plan (KNAP) and active participation in mainstreaming global commitments to country development plans have aimed to strengthen MNG. Nevertheless, MNG gaps persist, particularly in translating political commitments into effective implementation and measurable outcomes [16,17]. Assessing the status of MNG and factors that influence its performance is essential for informing policy and strengthening governance effectiveness. It is crucial to move beyond descriptive reporting across the six governance domains toward a systematic diagnosis of constraints and identification of strategic entry points for improvement [[17], [18], [19]].

One approach is to organize MNG domains into 3 functional themes which comprise *1*) foundational commitment, *2*) enabling capacity and resources, and *3*) institutionalized mechanisms for action. This lens provides a structured framework for assessing governance performance and prioritizing reforms. Understanding the current status of the three MNG themes and their role in shaping nutrition MNG is vital for generating actionable policy recommendations. Such insights are particularly important as the government of Kenya and stakeholders seek to accelerate progress toward national and global nutrition targets and strengthen devolved governance systems [7,20]. This study therefore aimed to generate evidence on the effectiveness of MNG over the past decade and to analyze how the 3 governance themes influence nutrition governance outcomes. The study was guided by 3 research questions: *1*) What is the current status of commitment, capacity, and mechanisms for action for MNG in Kenya, and how have these themes evolved over the past decade? *2*) To what extent does the 3 themes explain variations in MNG status in 2023? *3*) And to what extent do changes in the 3 themes explain improvements in MNG between 2012 and 2023?

### Methods

#### Links to the larger study

This study was nested within a larger mixed-methods research study that examined the status of MNG, its determinants, and its association with nutrition outcomes in Kenya. The parent study is published in Korir (2025) and is available at https://hdl.handle.net/2346/103021. The parent study established that overall MNG improved significantly from 58.4% in 2012 to 71.1% in 2023 (*P* < 0.01), with the strongest gains observed in political commitment, policy coherence, and multisectoral coordination, whereas comparatively weaker performance persisted in financing, accountability, transparency, and implementation capacity. It further demonstrated that higher MNG scores were associated with ministries that prioritized nutrition and with institutional affiliation to the Ministry of Health (MoH) (*P* < 0.05). Qualitative findings corroborated these patterns while highlighting persistent constraints, including inadequate financing, donor dependence, and limited institutional capacity.

Building on parent study findings, the present study constitutes an analytical extension rather than a replication. Although the parent study primarily described MNG status and associated factors, it did not systematically examine how different governance components jointly shape governance outcomes. To address this gap, the current study reorganizes the six MNG domains into three functional themes comprising commitment, capacity and resources, and mechanisms for action and applies inferential and relative importance analyses to assess their contribution to variations in MNG status and observed improvements over time. This approach provides a more integrated and explanatory perspective on governance performance which advances understanding beyond descriptive assessment toward identifying priority leverage points for strengthening MNG in Kenya.

### Study design

A retrospective cross-sectional design was used, whereby respondents assessed current MNG conditions in 2023 when data were collected and retrospectively assessed conditions in 2012 to examine changes in the preceding decade. It is important to note that the study does not assume continuity of governance conditions over time; rather, it captures 2 assessments of MNG at distinct time points. Year 2012 was purposefully selected since it marked a critical policy juncture in Kenya’s nutrition landscape. The 2 key frameworks, the National Food and Nutrition Security Policy and the first KNAP, were adopted in 2012. In addition, Kenya strengthened its commitment to global nutrition initiatives by endorsing the World Health Assembly (WHA) nutrition targets and joining the Scaling Up Nutrition (SUN) Movement around the same period [7,22,23]. These widely recognized policy milestones provided a structured reference point for retrospective assessment. In addition, the study targeted respondents in active roles with substantial sectoral experience, which supported informed recall. Moreover, the potential recall bias was mitigated through the inclusion of a “don’t know” response option in the questionnaire and the focus on institutional-level processes rather than individual-level events. Given that the 2023 assessment reflects governance conditions during and after the COVID-19 pandemic, respondent ratings inherently capture pandemic-related disruptions and adaptations within MNG systems.

### Ethical approval and consent

This study was conducted according to the guidelines laid down in the Declaration of Helsinki, and all procedures involving research study participants were approved by the relevant ethics committees. Ethical approvals were obtained from the Texas Tech University Institutional Review Board (IRB2023-493) and the Amref Ethics and Scientific Review Committee (ESRC P1443/2023). Authorization to conduct the research was granted by the National Commission for Science, Technology and Innovation in Kenya (NACOSTI/P/23/27685). Written informed consent was obtained from all participants before data collection, including consent for the publication of study results.

### Study area and target population

This study was conducted at the national level in Kenya, focusing on institutions directly involved in nutrition governance. The target population comprised government program managers/directors and program officers working within line ministries responsible for nutrition-related policy development, implementation, coordination, and monitoring. It also included program managers/directors and program officers from nongovernmental organizations (NGOs) and partner institutions providing technical or implementation support to the nutrition sector at the national level. These categories of respondents were selected because MNG processes are primarily operationalized through individuals occupying programmatic and policy-relevant roles, including decision-making, implementation coordination, and accountability functions [4,18,19]. Putting an emphasis on actors embedded within these institutional structures enabled the study to capture informed assessments of governance processes rather than general perceptions.

### Sampling design and inclusion/exclusion criteria

A structured multistage sampling approach was employed to identify and recruit respondents. First, a comprehensive mapping of eligible institutions and actors involved in nutrition governance at the national level was conducted in collaboration with the Division of Family Wellness, Nutrition and Dietetics within the MoH, which coordinates nutrition activities in Kenya. This process generated a defined sampling frame of relevant actors (*N* = 124), representing a finite population of individuals occupying key roles in nutrition policy and program implementation across government and nongovernmental sectors.

Inclusion criteria required that respondents hold positions as program managers/directors or program officers within departments, divisions, or units directly engaged in nutrition planning, implementation, or coordination. For NGOs, inclusion was limited to those actively supporting nutrition programs at the national level. Exclusion criteria applied to individuals in administrative or support units (e.g., human resources and legal services) and organizations without a direct role in nutrition programming.

To ensure representation across the governance system, the sampling frame was organized into 3 functional quotas: *1*) government staff from primary line ministries (*n* = 52), *2*) government staff from secondary ministries (*n* = 11), and *3*) nongovernmental actors supporting the nutrition sector (*n* = 61). These quotas reflect the institutional distribution of actors within Kenya’s MNG system rather than proportional sampling. From this sampling frame, 90% of eligible respondents within each category were targeted for participation, yielding a sample size of 112. This approach reflects a near-census of the identified governance actors and is appropriate for studies examining institutional processes within a defined system, where the objective is comprehensive coverage rather than statistical generalization [18,21].

### Data collection instrument

A structured, closed-ended questionnaire adapted from the validated Nutrition Governance Index (NGI) tool [18] and aligned with existing MNG frameworks [4,19,24] was used. The 6 core domains of the NGI were retained. Selected items were refined to improve clarity and align with the Kenyan context. Additional adjustments were made to address previously noted weaknesses in capacity-related domains. The final instrument consisted of 27 interrelated items or questions, each measured on a 5-point Likert scale (1 = strongly disagree to 5 = strongly agree). The items covered 6 MNG domains: political commitment and leadership, policy coherence and coordination, transparency and accountability, financing, capacity, and results measurement and monitoring (Table 1). To assess change over time, the questionnaire was structured to capture responses at 2 time points: 2012 and 2023. The year 2012 was selected as a policy reference point marking the adoption of key national nutrition frameworks as described in section “Study design.” Respondents also provided background information, including role, institutional affiliation, and years of experience.

**TABLE 1:** MNG domains and corresponding items

A. Political commitment, authority and leadership 1. There is commitment by the ministry leadership to strengthen nutrition in this ministry. 2. There is clear leadership in this ministry in terms of defining what needs to be done to scale up nutrition. 3. The ministry leadership is committed to improving institutional and operational procedures. 4. There is adequate technical and operational support from the leadership of the ministry to improve nutrition. 5. There are active nutrition champions in this ministry.
B. Policy coherence and coordination 1. Plans in the ministry are aligned to the NFSNP and KNAP. 2. Policies, strategies, plans, legislations, and other tools in the ministry incorporate nutrition adequately. 3. There is a request and commitment from other ministries to collaborate. 4. The roles and expectations of collaborating with other ministries on nutrition are clear. 5. There is adequate collaboration among various departments within this ministry. 6. There is adequate collaboration between this ministry and other ministries. 7. Information on nutrition plans and activities is shared with other ministries.
C. Transparency and accountability 1. The ministry has defined and transparent roles, expectations, and targets for nutrition actions. 2. Roles and expectations on nutrition are communicated regularly. 3. The ministry has mechanisms or incentives to appreciate and promote good performance.
D. Financing 1. Nutrition is formally taken into consideration in annual plans and budgets in the ministry. 2. The ministry allocates sufficient annual budget for nutrition activities.
E. Capacity 1. The ministry has adequate number of trained/skilled staff to effectively plan and implement. 2. Relevant ministry staff have adequate technical capacity to plan and implement. 3. The ministry has regular forums to strengthen the capacity to plan and implement.
F. Results measurement and monitoring 1. The ministry has adequate systems and processes to collect and report nutrition data. 2. The ministry has adequate capacity to monitor and evaluate nutrition-related plans. 3. The ministry regularly collects and reports nutrition information. 4. The information collected by the ministry is relevant to the needs. 5. Nutrition actions in this ministry are informed by the data/information that is collected. 6. Nutrition-related information collected by this ministry is shared with other ministries.

Each of the 27 questions or items was measured on a 5-point Likert scale (1 = strongly disagree; 2 = disagree; 3 = neutral; 4 = agree; 5 = strongly agree).

Abbreviations: KNAP, Kenya Nutrition Action Plan; MNG, Multisectoral Nutrition Governance; NFSNP, National Food and Nutrition Security Policy.

The data collection tool underwent a comprehensive pretest. Initial feedback was collected from faculty and doctoral students at Texas Tech University. After the initial feedback, a pilot test with 16 government and nongovernment respondents in Kenya was then conducted to assess reliability. Cronbach’s *α* for the 27 core items was 0.94, indicating high internal consistency [25]. Test–retest reliability over a 1–3 week period yielded a stability coefficient of 0.8 [26]. The survey was administered electronically using Qualtrics (Qualtrics International Inc., Provo, UT, USA; Version XM/os2, July 2023), which enabled structured data collection and secure data management.

### Computing MNG status and the 3 themes

The MNG status score was derived from the 27 Likert-type items (ranging from 1 to 5) using a hierarchical aggregation procedure. In this approach, item-level responses were first grouped by domain and averaged to generate scores for each of the 6 MNG domains. This step ensured that each domain contributed equally regardless of the number of items it contained. The resulting 6 domain means were then averaged to compute the overall MNG index for 2012 and 2023. Averaging rather than summing raw item scores normalized the scale and accommodated missing responses [27], thereby yielding a more consistent and interpretable measure of governance status [28].

To support analysis and synthesis at the theme level, the 6 original domains were consolidated into 3 composite themes aligned with the governance-to-action continuum. This consolidation was undertaken to reduce conceptual overlap across domains and to enable a more interpretable and policy-relevant analysis of how governance functions operate in combination. The 3 themes comprise: *1*) foundational commitment, comprising political commitment and policy coherence, which establish the policy environment necessary for prioritizing nutrition; *2*) enabling capacity and resources, encompassing sustainable financing and implementation capacity to operationalize policy intentions; and *3*) institutionalized mechanisms for action, including accountability systems and results measurement to facilitate continuous learning and performance improvement [4,19,29]. This framework provides a structured basis for assessing governance performance and prioritizing reform efforts. Moreover, it aligns with global strategies such as the UNICEF Nutrition Strategy (2020–2030), which underscores the critical role of enabling environments, institutional capacities, and delivery mechanisms in advancing sustainable nutrition outcomes [29].

### Statistical analyses

Before inferential analyses, data were tested for normality, linearity, homoscedasticity, and independence. Detailed diagnostic tests and results are provided in Supplemental Methods. The Shapiro–Wilk test assessed normality, whereas Levene’s test evaluated homogeneity of variances. The Durbin–Watson test was used to assess autocorrelation in regression residuals [30]. Data were analyzed using R (R Foundation for Statistical Computing, Vienna, Austria; Version 4.3.3). Descriptive statistics (means, SDs, frequencies, and percentages) summarized respondent characteristics and MNG scores for 2012 and 2023.

A paired Wilcoxon Signed-Rank test was used to compare commitment, capacity, and mechanisms for action between the 2 time points. Multiple linear regression models were used to assess the extent to which commitment, capacity, and mechanisms for action explained variations in MNG status in 2023 and improvements over the past decade. Statistical significance was set at *P* < 0.05.

## Results

### Survey respondents characteristics

The response rate for completing the survey was 77.4% (96 of 124). A response rate over 70% is considered robust in survey research targeting highly specialized target groups [31]. The high response rate was due to a clearly defined target population, the strong interest of the respondents in the subject matter of our research, and frequent follow-up to complete the survey. Out of the 96 respondents who completed the survey, representation was concentrated among individuals holding senior programmatic roles with (*n* = 60, 62.5%) being program managers and program directors. Respondents were predominantly (*n* = 89, 92.7%) affiliated with, or provided technical support to, primary line ministries, with Health and Agriculture Ministries dominating (*n* = 70, 73.0%). There were more respondents (*n* = 50, 52.1%) who worked for the government as compared with those who worked for NGOs (*n* = 46, 47.9%). In terms of professional experience, the larger proportion of respondents (*n* = 69, 71.9%) reported more than 10 years of engagement within their respective ministries, indicating a respondent pool with sustained exposure to MNG processes over the reference period. A comparison of available characteristics between respondents and nonrespondents (e.g., ministry affiliation, sector affiliation and year of experience) showed no meaningful differences, suggesting a limited risk of nonresponse bias.

### Status of MNG and its themes and their evolution over the past decade

**FIGURE 1:**
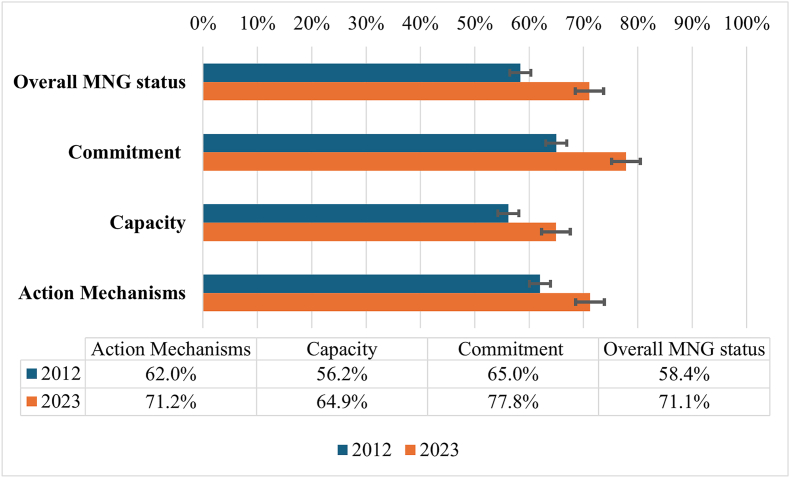
Mean percentage scores for overall multisectoral MNG and its composite themes (commitment, capacity, and mechanisms for action) in 2012 and 2023 (*n* = 96 paired responses). Scores derived from Likert-scale items and scaled 0%–100%, with error bars indicating 95% confidence intervals. MNG, Multisectoral Nutrition Governance.

Descriptive data analysis revealed improvements in the overall status of MNG and its core themes (commitment, capacity, and mechanisms for action) between 2012 and 2023. The mean MNG status index increased from 58.4% in 2012 to 71.1% in 2023, reflecting substantial progress in governance performance over the decade. Improvements were also observed across all 3 governance domains, indicating broad-based strengthening of MNG functions. Commitment to nutrition governance increased from 65.0% in 2012 to 77.8% in 2023, whereas capacity improved from 56.2% to 64.9%, and mechanisms for action increased from 62.0% to 71.2% during the same period (Figure 1).

Before assessing whether the mean change between the 2 time points was statistically significant, relevant assumptions were evaluated. Detailed diagnostic results are provided in Supplemental Methods. Diagnostic results indicated deviations from normality for some domains; therefore, for analytical consistency, the Wilcoxon Signed-Rank Test was applied across all domains as a nonparametric alternative. The Wilcoxon Signed-Rank Tests confirmed that these increases in MNG and the 3 themes were statistically significant, indicating consistent improvements across key governance functions. Specifically, the test results showed significant improvements in commitment (*V* = 3396, *P* < 0.01), capacity (*V* = 2723, *P* < 0.01), and mechanisms for action (*V* = 3055, *P* < 0.01). Effect size estimates based on the rank-biserial correlation indicated large practical significance, with values of 0.68 for commitment, 0.61 for capacity, and 0.67 for mechanisms for action. This suggests that the observed changes were not only statistically significant but also meaningful in practice (Table 2).

**TABLE 2:** Relative importance of governance themes in explaining the MNG status

Governance component	2012 (%)	2023 (%)	Wilcoxon V^1^	*P* value^2^	*r*_*b*_^3^	95% CI for mean diff.^4^
MNG status (overall)	58.4	71.1	3969	< 0.01	0.73	9.3, 15.8
Commitment	65.0	77.8	3396	< 0.01	0.68	7.4, 18.3
Capacity	56.2	64.9	2723	< 0.01	0.61	6.22, 11.5
Mechanisms for action	62.0	71.2	3055	< 0.01	0.67	7.05, 11.8

Abbreviations: CI, confidence interval; Diff., difference; MNG, Multisectoral Nutrition Governance; *r*_*b*_*,* Rank-biserial correlation; V, Wilcoxon signed rank test statistic.

1 Wilcoxon signed rank test.

2 Statistical significance set at *P* < 0.05.

3 Rank-biserial correlation used to estimate effect size.

4 95% CI for mean difference.

### Extent to which the 3 themes explain variations in current MNG status (2023)

All relevant statistical assumptions were tested and satisfied before conducting regression analyses. Detailed diagnostic results are provided in Supplemental Methods. The multiple linear regression model demonstrated that commitment, capacity, and mechanisms for action collectively explained 98.8% of the variance in the current MNG status, indicating that these governance themes strongly capture variations in MNG performance. Among the predictors, capacity emerged as the strongest, with a standardized regression coefficient (*β* = 0.51, *P* < 0.01), highlighting the central role of operational and resource-related factors in shaping governance outcome. Mechanisms for action also had a strong and significant influence (*β* = 0.47, *P* < 0.01), whereas commitment remained a significant but slightly less influential predictor (*β* = 0.39, *P* = 0.02), suggesting that while policy prioritization is important, its effects may depend on supporting capacity and institutional mechanisms. Relative importance analysis quantified the contribution of each theme to explained variance in MNG status. Capacity theme accounted for 35.5% of the variance [95% confidence interval (CI): 33.1%, 38.3%], followed by mechanisms for action theme at 33.2% (95% CI: 30.0%, 35.9%) and commitment theme at 31.2% (95% CI: 28.0%, 34.4%). Although capacity showed the largest point estimate, the overlapping CIs indicate that all 3 domains contributed comparably to variation in MNG status, suggesting no single dominant driver.

### Extent to which the 3 themes explain improvements in MNG over the past decade (2012*–*2023)

Linear regression analysis was also used to examine how changes in commitment, capacity, and mechanisms for action explained improvements in MNG between 2012 and 2023. The model accounted for 90.1% of the variance in governance improvements, indicating a strong explanatory power of the 3 governance themes. Similar to the findings for current MNG status, capacity change emerged as the most influential predictor (*β* = 0.50, *P* < 0.001), followed closely by mechanisms for action change (*β* = 0.48, *P* < 0.001). Commitment change had a moderate but still significant effect (*β* = 0.36, *P* = 0.003). Relative importance analysis showed that capacity change explained 34.7% of the variance (95% CI: 30.7%, 40.4%), mechanisms for action change accounted for 33.7% (95% CI: 27.5%, 39.6%), and commitment change explained 31.6% (95% CI: 24.0%, 37.6%).

The capacity theme exhibited a marginally greater numerical contribution; however, the CIs for all predictors overlapped, indicating no statistically meaningful differentiation among the 3 governance domains. This pattern implies that improvements in any of the domains were comparably associated with advances in MNG outcomes over the past decade, rather than being driven by a single domain. Further descriptive analysis of the 2 domains constituting the capacity theme demonstrated temporal improvement, albeit at different magnitudes. The financing domain increased from 51.4% in 2012 to 61.0% in 2023 (+9.6%), whereas the capacity and capability for implementing nutrition domain rose from 60.2% to 68.8% (+8.6%). Despite broadly similar gains, financing remained the lowest-performing domain in 2023. This persistent relative deficit suggests ongoing resource constraints and identifies financing as a continuing area of concern within the MNG system.

## Discussion

This study assessed the status and evolution of MNG in Kenya over the period from 2012 to 2023 while focusing on the role of commitment, capacity, and mechanisms for action in driving MNG. MNG status improved significantly from 58.4% in 2012 to 71.1% in 2023 (*P* < 0.01). Significant improvements were observed across the 3 MNG themes; commitment (*r*_*b*_ = 0.68), capacity (*r*_*b*_ = 0.61), and mechanisms for action (*r*_*b*_ = 0.67). Relative importance analysis indicated that the capacity theme accounted for the largest share of explained variance (35.5%), followed by mechanisms for action (33.2%) and commitment (31.2%); however, the overlapping CIs suggest that the 3 themes were comparably associated with variations in MNG status and improvements over the 2 time points. Examination of the domains within the capacity theme provided additional context. Although both implementation capability and financing improved, financing remained the lowest-performing domain, indicating persistent resource constraints. Taken together, these findings suggest that while governance progress reflects contributions across all 3 themes, the capacity theme most clearly represents the operational conditions through which policy commitments are translated into functioning multisectoral governance structures, consistent with prior governance scholarship [4,15].

The study period overlaps with the COVID-19 pandemic, which disrupted health and food systems, led to reallocation of public finances, and affected multisectoral coordination globally [32,33]. In Kenya, these disruptions likely influenced governance performance, particularly within the capacity domain, where financing and implementation systems were strained. The relatively lower performance of financing observed in this study may therefore partly reflect pandemic-related resource diversion and operational constraints. At the same time, the continued improvement in overall MNG suggests a degree of institutional resilience, whereby governance structures adapted to maintain coordination and policy momentum despite systemic shocks.

Kenya’s progress in MNG is consistent with increasing political attention to nutrition and efforts to institutionalize governance structures supporting multisectoral action [7,12]. The observed improvements reflect a stronger national prioritization of nutrition within development policies. This trajectory aligns with Kenya’s adoption of national policy frameworks and participation in global initiatives, including the Sustainable Development Goals, World Health Assembly nutrition targets, and the SUN Movement [1,22]. Although these commitments help establish policy direction and mobilize coordination, the findings indicate that commitment alone is insufficient for effective governance performance. Rather, improvements in governance appear to depend on the extent to which administrative and resource capacities enable implementation of agreed priorities. In this respect, commitment may initiate policy momentum, whereas capacity determines the extent to which that momentum can be operationalized within government systems [11,34]. The capacity theme was consistently associated with both current MNG status and improvements over the past decade. This finding underscores the importance of human, institutional, technical, and financial resources in enabling implementation of multisectoral nutrition policies [5,19,35]. In the absence of adequately trained staff and sufficient resources, policy commitments are unlikely to translate into sustainable MNG actions. Similar patterns have been described in other settings, such as Brazil, India, Nepal, and Ethiopia, where sustained improvements in nutrition governance were accompanied by investments in administrative coordination, sustainable financing, technical expertise, and enhanced institutional arrangements [4,18,36,37]. These examples suggest that governance progress is not solely a function of policy intent but depends on the capacity of public institutions to implement, coordinate, and sustain multisectoral activities. Strengthening such capacity supports continuity of nutrition governance processes and reduces vulnerability of nutrition initiatives to political or administrative transitions [11].

Mechanisms for action, including monitoring, accountability, and coordination platforms, were also associated with MNG outcomes. These mechanisms institutionalize governance practices by structuring how sectors and ministries plan, implement, and review activities, thereby supporting implementation and performance tracking [18]. In multisectoral settings, coordination platforms help align sectoral mandates and reduce fragmentation of actions across health, agriculture, social protection, and related sectors. When functioning effectively, they facilitate joint planning, shared reporting, and collective review of progress, promoting coherence in program delivery. However, their effectiveness depends on the availability of sufficient administrative and technical capacity to operate these systems and to use generated information for decision-making [38]. The findings therefore indicate that mechanisms for action are necessary for sustaining governance performance, but their practical utility is closely linked to the presence of capable institutions and personnel able to manage coordination and accountability processes.

Commitment remains essential for agenda setting and policy prioritization. The findings suggest that MNG performance depends on how commitments are institutionalized through administrative and operational systems [39,40]. In practice, commitment and capacity are closely related rather than independent. Sustained political and organizational commitment can enable the development of institutional structures, staffing, and financing, whereas established capacity allows commitments to be implemented consistently over time. The results therefore indicate not a hierarchy among themes, but a functional distinction, that is, commitment defines policy direction, whereas capacity reflects the system’s ability to operationalize agreed priorities. From this perspective, the comparatively higher contribution of the capacity theme reflects its role as the point at which political intent is translated into routine governance processes rather than evidence of statistical dominance.

The relative contributions of commitment, capacity, and mechanisms for action highlight the interdependence of these governance components. Progress in one area can reinforce advances in others, as political commitment can facilitate investment in institutional structures, whereas functioning mechanisms for coordination and accountability can, in turn, sustain commitment by demonstrating performance. Within this mutually reinforcing relationship, the capacity theme represents the administrative and resource conditions that enable governance processes to operate in practice. Rather than acting as an independent driver, capacity appears to function as the interface through which policy priorities and coordination arrangements are translated into routine governmental action [15,19]. Strengthening institutional capability and financing therefore supports not only implementation of multisectoral activities but also the effective functioning of accountability and coordination mechanisms over time.

These findings have practical implications for strengthening MNG. First, policy commitment should be accompanied by deliberate investments in operational capacity, particularly in staffing, administrative coordination, and predictable financing, as these conditions enable implementation of agreed multisectoral priorities [4,41]. Second, coordination and accountability mechanisms are most effective when supported by functional institutions capable of routine planning, reporting, and review; establishing platforms without the personnel and resources to operate them is unlikely to sustain governance performance. Third, governance reforms may benefit from a sequenced approach in which policy direction is followed by capacity development and subsequently reinforced through monitoring and accountability processes. An integrated strategy that aligns commitment, capacity, and mechanisms for action, with emphasis on strengthening operational capacity, is therefore more likely to sustain improvements in governance performance in resource-constrained settings.

This study has several strengths, including the use of a validated governance assessment tool and the application of rigorous statistical analyses, which enabled a systematic and quantitative assessment of changes in MNG. However, important limitations should be considered when interpreting the findings. The retrospective assessment of governance in 2012 relied on respondent recall and perceptions, which may introduce recall and response bias. In addition, the findings reflect respondents’ perceptions of changes in governance rather than directly observed institutional change and should be interpreted within this context. Although the use of policy reference points and experienced respondents supported informed recall, some degree of bias cannot be excluded. The design also captured governance at 2 time points and therefore cannot establish causal pathways linking specific policies or initiatives to observed changes. The national-level scope may also obscure variation across counties, where implementation arrangements, coordination practices, and accountability mechanisms often differ substantially. Furthermore, the study relied primarily on respondents involved in national nutrition activities, with limited representation of subnational implementation-level actors, which may reflect institutional perspectives more than frontline implementation experiences. Finally, the study period overlapped with the COVID-19 pandemic, which may have influenced governance processes, particularly in relation to financing and coordination, although these effects were not explicitly modeled. Future research would benefit from longitudinal designs, multilevel analyses incorporating county-level governance processes, and examination of interactions among sectors such as health, agriculture, finance, education, and nongovernmental actors. Such approaches, including triangulation with contemporaneous policy and administrative data, would strengthen inference on governance change over time [3].

In conclusion, this study provides evidence of Kenya’s progress in strengthening MNG and SUN actions over the past decade. Although all governance components remain important, the findings indicate that capacity (technical, institutional, and financial) represents the key interface through which commitment is translated into effective MNG. Sustaining progress will therefore require continued investment in operational capacity, particularly in predictable financing, skilled personnel, and functional coordination and administrative systems, alongside efforts to reinforce political commitment and maintain accountability mechanisms. Policymakers and practitioners should prioritize reforms that address all governance components while emphasizing the practical capabilities that enable implementation and support sustained improvements in MNG.

## Author contributions

The authors’ responsibilities were as follows – JKK, WO-T, WNG-W: conducted research; JKK: analyzed data; JKK, WNG-W: primary responsible for final content; and all authors: designed research, wrote the paper, read and approved the final manuscript.

## Data availability

Data described in the manuscript, code book, and analytic code will be made available upon request pending approval. For additional information, see our website at https://jn.nutrition.org/content/authorinfo/.

## Declaration of Generative AI and AI-assisted technologies in the writing process

The authors declare that no generative AI or AI-assisted technologies were used in the writing of this manuscript.

## Funding

The authors reported no funding received for this study.

## Conflict of interest

The authors report no conflicts of interest.
