# Supplementary material for: Operationalizing Multisectoral Nutrition Governance: Examining Commitment, Capacity, and Mechanisms for Action in Scaling Up Nutrition Actions in Kenya
Source: Curr Dev Nutr. 2026 Jun 20;10(7):109403. doi: 10.1016/j.cdnut.2026.109403 (PMC13379995; doi:10.1016/j.cdnut.2026.109403)
Supplement: multimedia component 1 [file mmc1.docx]

**Supplementary Material**

**A. Supplementary Methods**

### *Diagnostic Tests*

Prior to inferential analyses, data were tested for normality, linearity, homoscedasticity, and independence. The Shapiro-Wilk test assessed normality, while Levene’s test evaluated homogeneity of variances. The Durbin-Watson test was used to assess autocorrelation in regression residuals (Field, 2013).

Prior to assessing whether the mean change between the two time points was statistically significant, the assumptions underlying were evaluated. All relevant assumptions were assessed prior to analysis and were adequately met or addressed. To assess assumptions for the paired-samples t-test, we examined the distribution of the difference scores using the Shapiro-Wilk test, skewness, kurtosis, histogram, and Q-Q plots. For the Commitment domain, the Shapiro-Wilk test was not significant (W = 0.977, p = 0.102), indicating no strong evidence of non-normality. Skewness (0.24) and kurtosis (4.09) also fell within acceptable ranges, suggesting approximate normality. However, for Capacity (W = 0.958, p = 0.004) and Action (W = 0.961, p = 0.006), the Shapiro-Wilk tests indicated significant deviations from normality. Although the skewness and kurtosis values for these domains (Capacity: skewness = -0.50, kurtosis = 5.19; Action: skewness = 0.66, kurtosis = 4.21) suggested only moderate departures, the histograms and Q-Q plots displayed notable deviations, particularly at the tails. Given these findings and for analytical consistency, the Wilcoxon Signed-Rank Test was used across all domains as a non-parametric alternative.

All relevant statistical assumptions were tested and satisfied prior to conducting regression analysis. The Durbin-Watson tests confirmed no significant autocorrelation of residuals, with p-values greater than 0.05. Multicollinearity was not a concern, as all variance inflation factor (VIF) values were below 5. The Shapiro-Wilk tests and Q-Q plots confirmed normality of residuals (*p* > 0.05) , and the Breusch-Pagan tests indicated no evidence of heteroscedasticity (*p* > 0.05).

**B. Data Collection Tool/MNG Questionnaire**

| ***Purpose of the questionnaire: To collect information on the status of Multisectoral Nutrition Governance (MNG) in Kenya*** | | | |
| --- | --- | --- | --- |
| **Target group**  ***(i) Government staff -*** working in line ministries as program directors/managers and program officers.  ***(ii) Non-government*** stakeholders program directors/managers and program officers supporting the government to advance the nutrition agenda in Kenya *(development partners/donors, United Nations (UN) agencies, Civil Society Organizations (CSOs), private academic and research institutions and private sector partners among others).* | | | |
| **Instructions for completing the questionnaire**   1. The questions will ask about the ministry, sector, division or unit you work for, or support. 2. Take time to read each question carefully and make sure you understand what is being asked. 3. For each question, you will be required to select a response on (i) the present/current situation, and (ii) comparison of the current situation to the situation in 2012 when the National Food Security and Nutrition policy (NFNSP) and the first Kenya National Nutrition Action Plan (KNAP) were adopted. 4. Select the answer that best reflects your experience or opinion. You will have the chance to strongly agree/agree/neutral/disagree/strongly disagree with the statement. 5. Provide honest responses to each question, even if the answer may not reflect positively on your work or support. 6. Try your best to answer every question on the questionnaire, even if you are unsure of the answer. If there is a question you cannot answer, select the option that best reflects your level of uncertainty. | | | |
| Sex □ Male □ Female | | | |
| Position | | | |
| Government staff □ Yes □ No | | | |
| Non-governmental staff □ Yes □ No | | | |
| Which one best describes your role in your organization | | 1. Policy maker and/or decision maker  2. Program manager and/or program officer  3. Others (specify) | |
| If government staff which ministry do you represent (choose one only) | | 1. Health  2. Agriculture  3. Education (includes government academia)  4. Labour and Social Protection  5. Water and Sanitation  6. Treasury and Economic Planning  7. Public Service and Gender  8. Trade, Investments and Industry  9. Devolution and Planning  10. Others (specify) | |
| If non-government staff which category best represents your organization (choose one only) | | 1. Development partners/donors  2. United Nations agencies  3. Civil Society Organizations  4. Private sector  5. Academia (private research and academic institutions) | |
| If non-government which ministry do you primarily support to deliver nutrition actions (choose one only) | | 1. Health  2. Agriculture  3. Education (includes government academia)  4. Labour and Social Protection  5. Water and Sanitation  6. Treasury and Economic Planning  7. Public Service and Gender  8. Trade, Investments and Industry  9. Devolution and Planning  10. Others (specify) | |
| **MNG domains and items/variables** | | **Current situation**  **(2023)** | **Is the current situation similar to 2012 (when NFSNP and KNAP were adopted)** |
| **A. Political commitment, authority and leadership** | | | |
| 1 | The leadership in this ministry considers nutrition as a priority to be addressed | *5.Strongly agree*  *4.Agree*  *3.Neutral*  *2.Disagree*  *1.Strongly disagree*  *98.Don’t know* | *5.Strongly agree*  *4.Agree*  *3.Neutral*  *2.Disagree*  *1.Strongly disagree*  *98.Don’t know* |
| 2 | There is commitment by the ministry leadership to strengthen nutrition in this ministry | *5.Strongly agree*  *4.Agree*  *3.Neutral*  *2.Disagree*  *1.Strongly disagree*  *98.Don’t know* | *5.Strongly agree*  *4.Agree*  *3.Neutral*  *2.Disagree*  *1.Strongly disagree*  *98.Don’t know* |
| 3 | There is clear leadership in this ministry in terms of defining what needs to be done to scale up nutrition | *5.Strongly agree*  *4.Agree*  *3.Neutral*  *2.Disagree*  *1.Strongly disagree*  *98.Don’t know* | *5.Strongly agree*  *4.Agree*  *3.Neutral*  *2.Disagree*  *1.Strongly disagree*  *98.Don’t know* |
| 4 | The ministry leadership is committed to improving institutional and operational procedures to effectively deliver nutrition actions and commitments | *5.Strongly agree*  *4.Agree*  *3.Neutral*  *2.Disagree*  *1.Strongly disagree*  *98.Don’t know* | *5.Strongly agree*  *4.Agree*  *3.Neutral*  *2.Disagree*  *1.Strongly disagree*  *98.Don’t know* |
| 5 | There are active nutrition champions in this ministry (i.e., an influential people making compelling case for more attention and resources for nutrition actions) | *5.Strongly agree*  *4.Agree*  *3.Neutral*  *2.Disagree*  *1.Strongly disagree*  *98.Don’t know* | *5.Strongly agree*  *4.Agree*  *3.Neutral*  *2.Disagree*  *1.Strongly disagree*  *98.Don’t know* |
| 6 | There is adequate technical and operation support from the leadership for implementing my roles and responsibilities | *5.Strongly agree*  *4.Agree*  *3.Neutral*  *2.Disagree*  *1.Strongly disagree*  *98.Don’t know* | *5.Strongly agree*  *4.Agree*  *3.Neutral*  *2.Disagree*  *1.Strongly disagree*  *98.Don’t know* |
| **B. Policy coherence and coordination** | | | |
| 1 | Plans in the ministry are aligned to the National Food Security and Nutrition Policy (NFSNP) and the Kenya National Nutrition Action Plan (KNAP) | *5.Strongly agree*  *4.Agree*  *3.Neutral*  *2.Disagree*  *1.Strongly disagree*  *98.Don’t know* | *5.Strongly agree*  *4.Agree*  *3.Neutral*  *2.Disagree*  *1.Strongly disagree*  *98.Don’t know* |
| 2 | Policies, strategies, plans, legislations and other tools in the ministry incorporate nutrition adequately | *5.Strongly agree*  *4.Agree*  *3.Neutral*  *2.Disagree*  *1.Strongly disagree*  *98.Don’t know* | *5.Strongly agree*  *4.Agree*  *3.Neutral*  *2.Disagree*  *1.Strongly disagree*  *98.Don’t know* |
| 3 | Nutrition is taken into consideration in annual plans and budgets in the ministry | *5.Strongly agree*  *4.Agree*  *3.Neutral*  *2.Disagree*  *1.Strongly disagree*  *98.Don’t know* | *5.Strongly agree*  *4.Agree*  *3.Neutral*  *2.Disagree*  *1.Strongly disagree*  *98.Don’t know* |
| 4 | There is demand from other ministries to collaborate on nutrition related issues | *5.Strongly agree*  *4.Agree*  *3.Neutral*  *2.Disagree*  *1.Strongly disagree*  *98.Don’t know* | *5.Strongly agree*  *4.Agree*  *3.Neutral*  *2.Disagree*  *1.Strongly disagree*  *98.Don’t know* |
| 5 | The roles and expectations on collaborating with other ministries on nutrition are clear | *5.Strongly agree*  *4.Agree*  *3.Neutral*  *2.Disagree*  *1.Strongly disagree*  *98.Don’t know* | *5.Strongly agree*  *4.Agree*  *3.Neutral*  *2.Disagree*  *1.Strongly disagree*  *98.Don’t know* |
| 6 | There is adequate collaboration among various departments within this ministry in addressing nutrition issues | *5.Strongly agree*  *4.Agree*  *3.Neutral*  *2.Disagree*  *1.Strongly disagree*  *98.Don’t know* | *5.Strongly agree*  *4.Agree*  *3.Neutral*  *2.Disagree*  *1.Strongly disagree*  *98.Don’t know* |
| 7 | There is adequate collaboration between this ministry and other ministries (e.g., health, education, social protection etc.) in addressing nutrition issues | *5.Strongly agree*  *4.Agree*  *3.Neutral*  *2.Disagree*  *1.Strongly disagree*  *98.Don’t know* | *5.Strongly agree*  *4.Agree*  *3.Neutral*  *2.Disagree*  *1.Strongly disagree*  *98.Don’t know* |
| 8 | Information on nutrition plans and activities is shared with other ministries | *5.Strongly agree*  *4.Agree*  *3.Neutral*  *2.Disagree*  *1.Strongly disagree*  *98.Don’t know* | *5.Strongly agree*  *4.Agree*  *3.Neutral*  *2.Disagree*  *1.Strongly disagree*  *98.Don’t know* |
| **C. Transparency and accountability** | | | |
| 1 | The ministry has defined and transparent roles, expectations and targets on nutrition actions | *5.Strongly agree*  *4.Agree*  *3.Neutral*  *2.Disagree*  *1.Strongly disagree*  *98.Don’t know* | *5.Strongly agree*  *4.Agree*  *3.Neutral*  *2.Disagree*  *1.Strongly disagree*  *98.Don’t know* |
| 2 | Roles and expectations on nutrition are communicated regularly (monthly, quarterly or annually) | *5.Strongly agree*  *4.Agree*  *3.Neutral*  *2.Disagree*  *1.Strongly disagree*  *98.Don’t know* | *5.Strongly agree*  *4.Agree*  *3.Neutral*  *2.Disagree*  *1.Strongly disagree*  *98.Don’t know* |
| 3 | The ministry has mechanisms or incentives (e.g., rewards and recognition) to appreciate and promote good performance in meeting nutrition targets | *5.Strongly agree*  *4.Agree*  *3.Neutral*  *2.Disagree*  *1.Strongly disagree*  *98.Don’t know* | *5.Strongly agree*  *4.Agree*  *3.Neutral*  *2.Disagree*  *1.Strongly disagree*  *98.Don’t know* |
| **D. Financing** | | | |
| 1 | Nutrition is formally taken into consideration in annual plans and budgets in the ministry | *5.Strongly agree*  *4.Agree*  *3.Neutral*  *2.Disagree*  *1.Strongly disagree*  *98.Don’t know* | *5.Strongly agree*  *4.Agree*  *3.Neutral*  *2.Disagree*  *1.Strongly disagree*  *98.Don’t know* |
| 2 | The ministry has dedicated budget line for nutrition activities | *5.Strongly agree*  *4.Agree*  *3.Neutral*  *2.Disagree*  *1.Strongly disagree*  *98.Don’t know* | *5.Strongly agree*  *4.Agree*  *3.Neutral*  *2.Disagree*  *1.Strongly disagree*  *98.Don’t know* |
| 3 | The ministry allocates sufficient annual budget for nutrition activities | *5.Strongly agree*  *4.Agree*  *3.Neutral*  *2.Disagree*  *1.Strongly disagree*  *98.Don’t know* | *5.Strongly agree*  *4.Agree*  *3.Neutral*  *2.Disagree*  *1.Strongly disagree*  *98.Don’t know* |
| **E. Capacity and capability** | | | |
| 1 | The ministry has adequate number of trained/skilled staff to effectively plan and implement nutrition activities | *5.Strongly agree*  *4.Agree*  *3.Neutral*  *2.Disagree*  *1.Strongly disagree*  *98.Don’t know* | *5.Strongly agree*  *4.Agree*  *3.Neutral*  *2.Disagree*  *1.Strongly disagree*  *98.Don’t know* |
| 2 | Relevant ministry staff have adequate technical capacity to plan and implement nutrition activities | *5.Strongly agree*  *4.Agree*  *3.Neutral*  *2.Disagree*  *1.Strongly disagree*  *98.Don’t know* | *5.Strongly agree*  *4.Agree*  *3.Neutral*  *2.Disagree*  *1.Strongly disagree*  *98.Don’t know* |
| 3 | The ministry has regular (monthly, quarterly or annually) forums to strengthen capacity to plan and implement nutrition actions | *5.Strongly agree*  *4.Agree*  *3.Neutral*  *2.Disagree*  *1.Strongly disagree*  *98.Don’t know* | *5.Strongly agree*  *4.Agree*  *3.Neutral*  *2.Disagree*  *1.Strongly disagree*  *98.Don’t know* |
| **F. Results measurement and monitoring** | | | |
| 1 | The ministry has adequate systems and processes to collect and report nutrition data/information as stipulated in the annual plans | *5.Strongly agree*  *4.Agree*  *3.Neutral*  *2.Disagree*  *1.Strongly disagree*  *98.Don’t know* | *5.Strongly agree*  *4.Agree*  *3.Neutral*  *2.Disagree*  *1.Strongly disagree*  *98.Don’t know* |
| 2 | The ministry regularly (monthly, quarterly or annually) collects and reports nutrition information | *5.Strongly agree*  *4.Agree*  *3.Neutral*  *2.Disagree*  *1.Strongly disagree*  *98.Don’t know* | *5.Strongly agree*  *4.Agree*  *3.Neutral*  *2.Disagree*  *1.Strongly disagree*  *98.Don’t know* |
| 3 | The information collected by the ministry is relevant to the needs and impactful in improving nutrition actions | *5.Strongly agree*  *4.Agree*  *3.Neutral*  *2.Disagree*  *1.Strongly disagree*  *98.Don’t know* | *5.Strongly agree*  *4.Agree*  *3.Neutral*  *2.Disagree*  *1.Strongly disagree*  *98.Don’t know* |
| 5 | Nutrition actions in this ministry is informed by the data/information that is collected | *5.Strongly agree*  *4.Agree*  *3.Neutral*  *2.Disagree*  *1.Strongly disagree*  *98.Don’t know* | *5.Strongly agree*  *4.Agree*  *3.Neutral*  *2.Disagree*  *1.Strongly disagree*  *98.Don’t know* |
| 4 | Nutrition related information collected by this ministry is shared with other ministries to promote collaboration and joint accountability | *5.Strongly agree*  *4.Agree*  *3.Neutral*  *2.Disagree*  *1.Strongly disagree*  *98.Don’t know* | *5.Strongly agree*  *4.Agree*  *3.Neutral*  *2.Disagree*  *1.Strongly disagree*  *98.Don’t know* |
